# Increased Risk of COVID-19-Related Deaths among General Practitioners in Italy

**DOI:** 10.3390/healthcare8020155

**Published:** 2020-06-03

**Authors:** Alberto Modenese, Fabriziomaria Gobba

**Affiliations:** Department of Biomedical, Metabolic and Neural Sciences, Università degli Studi di Modena e Reggio Emilia, 41125 Modena, Italy; fabriziomaria.gobba@unimore.it

**Keywords:** general practitioners, novel coronavirus, occupational medicine

## Abstract

To date, data on COVID-19-related death cases among physicians from different medical specialties are incomplete and scattered. In Italy, available data highlight that general practitioners (GPs) are, apparently, the most heavily affected group. Indeed, they currently represent 44.1% of the total COVID-19 related death cases occurred among physicians, whereas they constitute about 15% of the total number of doctors. This high proportion is most likely the consequence of a work-related contagion happening especially during the first weeks of the epidemic, and persisting also in the following weeks, after the national lockdown. There are various reasons for these higher contagion rates: GPs perform a lot of medical examinations daily, usually in close contact with patients. Especially at the beginning of the epidemic, GPs might have had scant information on the specific safety procedures for the prevention of SARS-CoV-2 transmission (e.g., there was limited knowledge on the possibility of contagions deriving from asymptomatic patients) and, moreover, the availability of personal protective equipment was insufficient. Furthermore, the risk of infection is highly increased by the virus’ characteristics, like its survival for several hours/days on different surfaces and its persistence in the air after an aerosolization process, with possibilities to be transmitted over distances longer than two meters. Following these observations, and considering the high cost in term of GPs’ lives, the COVID-19 pandemic will probably revolutionize the approach to patients in general practice. Clear and effective guidelines are absolutely and urgently needed for the refinement of adequate measures to prevent SARS-CoV-2 infections among GPs.

## 1. Introduction

Healthcare workers (HCW) have a high occupational risk related to SARS-CoV-2 infection [1]. Italian data show that, currently, 11.9% of all the diagnosed COVID-19 cases (27,439 out of 230,414 diagnoses) occurred in HCW [2]. This high risk is not unexpected, and was also observed during the SARS and MERS outbreaks, respectively in 2003 and 2015.

Specific procedures for the protection of HCW have been proposed by authoritative organizations, such as the World Health Organization (WHO) [3], the European Centre for Disease Prevention and Control [4] and the Centre for Disease Control and Prevention (CDC) [5]. Nevertheless, available data call into question the effectiveness of current preventive procedures and support the need for further development that, in the era of precision medicine, should be more tailored to the specific activities performed. In this context, an interesting aspect likely to provide useful insight is knowledge on variations in disease occurrence among physicians practicing specific medical specialties. Significant differences are likely, for example, in the type and modality of contact with patients, in the environment where contacts occur, as well as in the medical procedures applied. A group with various specific peculiarities is certainly that of family physicians/general practitioners (GPs), representing one of the front lines of the war against COVID-19. GPs have been visiting an overwhelming number of patients, often directly at their homes, with scarce possibilities, if any, to control the work environment. Especially during the first phases of the outbreak, they were unaware of the presence of SARS-CoV-2-infected patients, with an incomplete knowledge of the risk, of the adequate preventive procedures to be applied and, possibly, also with an insufficient/inadequate availability of personal protective equipment (PPE) [6]. 

## 2. Discussion: The Italian Data on COVID-19-Related Deaths among General Practitioners and Other Physicians

**Figure 1:**
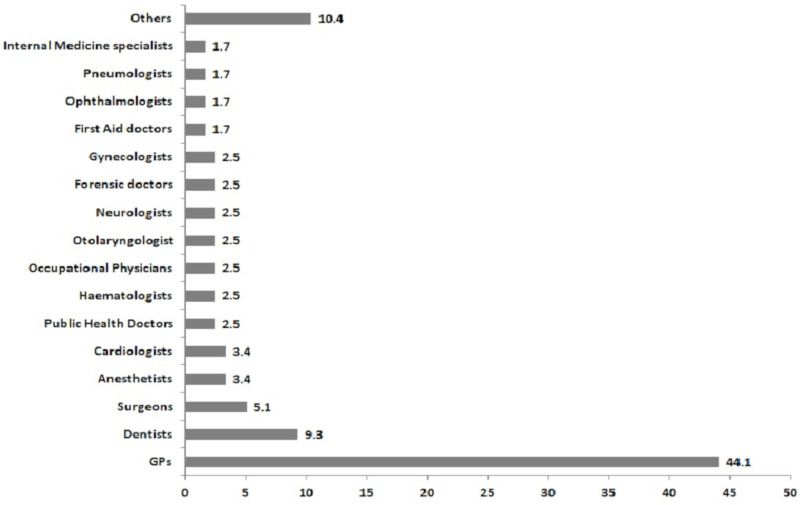
Percentages of physicians grouped according to their medical specialty among the 118 COVID-19-related death cases occurred in Italy between March and April 2020.

Data on specific COVID-19 contagion among GPs is currently scant, but, in Italy, one of the countries with the highest number of COVID-19 cases and mortality, at least some mortality data are available. In fact, the official webpage of the Italian Federation of the Colleges of Physicians (FNOMCEO) is publishing and updating daily a list of the Italian physicians that have died as a consequence of SARS-CoV-2 infection, also reporting their medical specialty [7]. As of the 30 April 2020, 156 physicians had died. All physicians who died due to COVID-19 are reported in this list, including both active and retired physicians. For this reason, in order to have a more reliable overview of the situation, we have excluded all cases of deaths which occurred in physicians over 75 years old (private practice is common for some years after formal retirement, which usually happens between 67 and 70 years). After this exclusion, the number of physician deaths due to COVID-19 which occurred in Italy by the 30 April 2020 is reduced to 118. During the following weeks of May (up to 26 May 2020) another eight deaths were reported, of which one was a GP and seven were physicians with other medical specialties. By 30 April 2020, there were 52 GPs—by far the most represented medical specialty—contributing 44.1% of the total number of COVID-19-related deaths among Italian physicians (Figure 1). GPs are followed by dentists and surgeons, representing, respectively, 9.3% and the 5.1% of all deaths, and then by anesthetists and cardiologists, both contributing four cases, corresponding to 3.4% (Figure 1).

**Figure 2:**
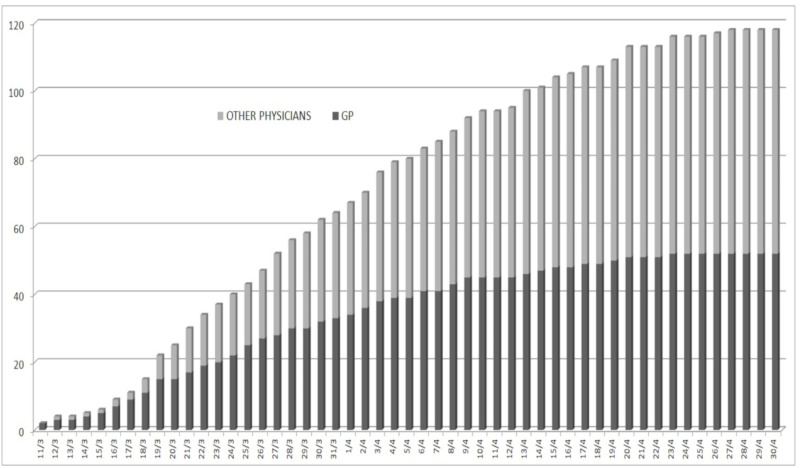
Daily cumulative increase of COVID-19-related-death cases among Italian physicians since the first reported death on the 11 March 2020 and up to the 30 April 2020.

The mean age of the physicians is similar in GPs and other medical specialties (66.5 vs 66.2 y/o) and is lower compared to the mean age of those who have died in the general population (79.5 years [8]), supporting the specificity of the contagion in these groups. The first Italian case of COVID-19 in Italy was diagnosed in the Lombardy region on 21 February, schools were closed on 4 March, and since the second week of March the majority of work activities have been locked down, until 18 May. The first cases of death among Italian physicians were reported on the FNOMCEO website on 11 March 2020 in two GPs practicing in the Lombardy region. Nevertheless, it should not be excluded that some cases may have occurred even before that date. At the end of April, we were at the eighth week of lockdown in Italy: Figure 2 shows the daily cumulative increase in death cases among physicians since the first case reported, reporting the trend of the 118 death cases in these eight weeks of national lockdown. It should be noted that, up to the 2nd of April, GP deaths were more common than deaths reported in other medical specialties, reaching 50% at the beginning of April; after that, the rate remained at 45%. In the second half of April, the proportion reached the current estimate, 44.1% (Figure 2). However, even if decreasing, this proportion is still relevantly higher, approximately three times greater than the overall ratio of GPs to physicians practicing other medical specialties in Italy, which can be estimated at around 15% according to the Italian national Institute of Statistics (ISTAT) [9], suggesting the persistence of a higher risk.

This difference between the proportion of GPs to other physicians (using currently available occupational data) and the deaths related to COVID-19 indicates a relevantly high occupational risk for GPs. Nevertheless, further well-designed studies will be needed to collect more precise data reporting COVID-19 prevalence, incidence and mortality rates among different medical specialties and hospital departments. Such studies will allow the identification of specific occupational hazards and possible increased risk of infection and mortality among various categories of HCWs. Considering the currently available Italian data, the trend in deaths suggests that the work-related contagion of GPs was more critical during the first weeks of the epidemic in Italy, with a slight decrease in more recent days, even if the proportion is still very high for GPs. The relatively high number of death cases among GPs deserves some examination. Considering the SARS-CoV-2 incubation period and the period elapsed between symptom onset and death, it can be said that many of GPs became infected in February or the first week of March, when in Italy there was still a scant awareness of the risk related to COVID-19 and, in general, risk perception was largely lower compared to the weeks after the national lockdown. Moreover, February and early March in Italy is a period of the year when GPs perform a lot of medical examinations of patients with influenza-like symptoms. Recent data have confirmed that SARS-CoV-2 salivary viral load is particularly high during the first week after symptom onset [10], and viral RNA has been documented in throat swabs for more than 40 days, with high titers in the saliva [11]. Furthermore, especially at the beginning of the epidemic, the exponential increase in cases over a few days did not give GPs enough time to adopt adequate safety procedures to visit the patients and to be appropriately informed on the most effective ways to prevent COVID-19 transmission. It should also be noted that the availability of personal protective equipment (PPE) was insufficient to allow the high number of visits requested, as recently reported in a survey from one of the Italian regions with the highest incidence of SARS-CoV-2 infections, Lombardy [12]. Another relevant point is that several studies documented SARS-CoV-2 infections in asymptomatic patients, i.e., persons not manifesting any symptoms inducing the suspicion of infection and, consequently, in no need of specific preventive measures [13]. These persons may have contributed to the transmission of infection to GPs, especially during the first weeks of the epidemic in Italy. In addition, it has to be considered that a medical examination usually implies a short distance between the patient and the doctor, but, even with reduced direct contact and extensive use of protections, there are additional problems, such as (a) the documented persistence of the pathogen on the surfaces of the clinic for up to several hours or a few days, depending on the type of material [14] and (b) recent evidence suggesting the possibility of viral transmission at distances greater than two meters, and its detection in the air up to three hours after aerosolization [15].

## 3. Conclusions

In conclusion, according to the abovementioned observations, and especially considering the serious loss of life among Italian GPs, the COVID-19 pandemic will probably revolutionize the approach to the patient in general practice [6,16]. The refining of adequate strategies and procedures to prevent COVID-19 infection among Italian GPs, as well as in the rest of the world, is crucial, and clear and effective guidelines are absolutely and urgently needed. The way the general practice system is organized in Italy may have played a role in the increased proportion of deaths among GPs. Even before of the COVID-19 outbreak, several critical points were observed: most of GPs are used to working single-handedly and, furthermore, there are difficulties in recruiting new workforces, making Italian family doctors quite an aged working category, with the 50% older than 55 y [17]. On average, the departments of general practice in Italy are open for less than 20 h per week, and doctors ensure continuity of care for out-of-hours emergencies at patients’ homes [17]. After the COVID-19 outbreak, many Italian scientific societies of general practice, such as the Italian Federation of Family Doctors (Federazione Italiana Medici di Famiglia—FIMMG) and the Italian Society of General Medicine (Società Italiana di Medicina Generale, SIMG), produced practical indications and operational guides for GPs in order to safely ensure care to their patients during the COVID-19 epidemic, adopting appropriate preventive measures to preserve their health [18,19]. Other important points to be stressed are the progressive activation, since the second half of March, in many Italian regions, of special medical units for the assistance of COVID-19 patients at home, called “USCA” (Unità Speciali di Continuità Assistenziale), as prescribed by a national decree [20], and the promotion of telemedicine procedures, when possible [21]. These new practices may have reduced the direct contact of GPs with patients actively infected with SARS-CoV-2.

It is not yet clear whether the slight decrease in the proportion of GPs among those physicians who died during the latest weeks of the epidemic in Italy may be attributed to these improvements in targeted prevention, or is only a reflection of the attenuation of the epidemic in Italy as a consequence of the lockdown. Nevertheless, the high proportion of deaths among GPs compared to other medical specialties, which persists, clearly indicates the need for further development of effective and, possibly, more tailored preventive measures.
